# Fetal Ventriculomegaly: A Review of Literature

**DOI:** 10.7759/cureus.22352

**Published:** 2022-02-18

**Authors:** Abdulelah A Alluhaybi, Khalid Altuhaini, Maqsood Ahmad

**Affiliations:** 1 Pediatric Neurosurgery, King Fahad Medical City/National Neuroscience Institute, Riyadh, SAU

**Keywords:** fetal ventriculomegaly, hydrocephalus, genetics, fetal ultrasound, fetal mri, cerebrospinal fluid, ventricular shunts

## Abstract

Fetal ventriculomegaly refers to ventricular enlargement that is diagnosed prenatally. It is one of the most common fetal anomalies. The diagnosis is made by ultrasound when the arterial diameter of the ventricle is more than 10 mm. Once it is diagnosed, further evaluation by detailed ultrasound, fetal MRI, and genetic studies is required. Prenatal surgical management of fetal ventriculomegaly is still limited and associated with high risks. Postnatal management is similar to the treatment of other types of hydrocephalus. Fetal ventriculomegaly is a heterogeneous condition with various etiologies and a wide spectrum of neurodevelopmental outcomes. The outcomes depend mainly on the severity of ventriculomegaly and associated structural abnormalities. This article aims to review the literature about various aspects of fetal ventriculomegaly.

## Introduction and background

Fetal ventriculomegaly is a ventricular dilatation that is detected on ultrasonography prenatally [1]. It occurs in two per 1000 live births [2,3] and is considered one of the most common fetal anomalies detected on ultrasonography during the second trimester [4]. It is categorized as mild (10-12 mm), moderate (13-15 mm), or severe (≥16 mm) according to ventricular diameter, measured on antenatal sonography or in utero MRI [5]. Fetal ventriculomegaly has multiple causes and produces a broad spectrum of neurodevelopmental outcomes [4]. Once it is identified, detailed ultrasonography should be performed to rule out other fetal structural anomalies [1]. Severe ventriculomegaly usually has obstructive causes and is associated with hydrocephalus [1]. Mild ventriculomegaly is often found incidentally and is considered benign, but it can be associated with genetic or structural abnormalities [1]. Isolated ventriculomegaly is defined as ventriculomegaly that is not associated with structural or genetic abnormalities identified on ultrasonography [6].

Postnatal management depends mainly on the rate of progression of ventriculomegaly and the development of hydrocephalus [4]. Neurodevelopmental outcomes and prognosis are influenced mainly by the severity of both ventriculomegaly and underlying structural or genetic abnormalities [7]. We review the causes, diagnosis, and treatment of fetal ventriculomegaly and the prognosis of affected patients.

## Review

The differential diagnosis of fetal ventriculomegaly is broad and includes normal variants when the size of the ventricle approaches 10 mm [1,8]. Fetal ventriculomegaly may be considered an indicator of chromosomal abnormalities, infection, or cerebral malformation, particularly when it is isolated [9]. Therefore, an extensive evaluation is necessary to determine the size of the ventricle and the underlying cause.

Etiology

The causes of fetal ventriculomegaly are divided into three main categories: cerebral parenchymal loss; obstructive causes; and overproduction of cerebrospinal fluid (CSF) [10]. Although isolated ventriculomegaly may be benign, chromosomal abnormalities are found in 2%-12% of cases [11]. Different causative processes include abnormal turnover of CSF, neuronal and migration disorders, and infection. Some cases are inherited X-linked or, in rare cases, autosomal recessive traits [9]. Intrauterine infections are found in approximately 5% of patients with fetal ventriculomegaly, and *Toxoplasma gondii*, rubella, cytomegalovirus, and herpes simplex virus infections are found in 10%-20% of those with severe isolated ventriculomegaly [12]. The most common structural causes of fetal ventriculomegaly include aqueductal stenosis, Chiari malformation type II, dysgenesis of the corpus callosum, and abnormalities of the posterior fossa [13,14]. Other causes include intraventricular hemorrhage and cortical development disorders, and other associated disorders include hydranencephaly and holoprosencephaly [15].

Diagnosis

Neuroimaging, including fetal ultrasonography and MRI, is crucial for the diagnosis of fetal ventriculomegaly. Each imaging modality has advantages and disadvantages [16]. When fetal ventriculomegaly is identified on screening antenatal ultrasonography, further workup with fetal ultrasonography or MRI is usually required [16].

Fetal ultrasonography

At the gestational age of 13-14 weeks, the lateral ventricles can be recognized; they change with the subsequent development of the cerebrum, and the choroid plexus usually serves as the landmark [17]. Although the overall ventricular diameter increases over time, the atrium remains stable during the second and third trimesters; for this reason, these are the preferred times for measurement [18]. The normal ventricular diameter in the fetal brain remains between 4.5 and 7.6 mm during the gestational ages of 15 and 40 weeks [8]. Most international guidelines mandate antenatal ultrasonography during the second trimester for measuring the diameter of the lateral ventricle in the axial transventricular plane. A diameter of ≥10 mm with at least 2.5 standard deviations above normal indicates ventriculomegaly and is considered abnormal [19].

Fetal MRI

Fetal MRI is an excellent diagnostic modality that enables the detection of additional abnormalities in approximately 50% of ventriculomegaly cases [20], such as agenesis of the corpus callosum, absence of the septum pellucidum, disorders of cortical development, and cerebrovascular abnormalities [21]. It is more sensitive than ultrasonography for detecting cortical malformation, hemorrhage, and parenchymal disorders [22]. Not all these findings may be clinically significant or associated with a poor prognosis, but early identification is helpful for parent counseling and directs further follow-up [23,24]. Measurements of ventricular diameter on axial MRI and ultrasonography differ by 1-2 mm [25]; however, measurements on coronal MRI and ultrasonography are highly similar [26].

Genetic testing

Karyotyping, used to identify aneuploidy and chromosomal abnormalities, is the standard method of genetic evaluation of fetal structural anomalies. An abnormal karyotype has been found in approximately 5% of cases of mild to moderate ventriculomegaly; trisomy 21 is the most common. Abnormal results of chromosomal microarray have been found in 10% of fetuses with abnormal findings on ultrasonography [27]. In 6% of fetuses with structural anomalies and normal karyotypes, microarray analysis has revealed clinically significant information [28]. Chromosomal microarray analysis is recommended as the first-tier test because it can reveal submicroscopic chromosomal abnormalities that are undetectable in conventional karyotyping [29].

Prenatal management

In the prenatal management of fetal ventriculomegaly, the efficacy of intrauterine ventricular shunting procedures is still limited [30]. In 1982, the International Fetal Medicine and Surgery Society established a voluntary international registry of fetal surgery. Thirty-nine cases of prenatal shunting have been reported; the fetal survival rate is 83%. Neurological disability has been severe and mild to moderate in >50% and 12% of the survivors, respectively; 35% have exhibited normal development [31-33]. Although early prenatal CSF procedures had unfavorable outcomes, the use of fetal CSF shunting has increased as a result of improvement in prenatal diagnosis and advances in fetal surgery [34].

Postnatal management

The first step in management is to identify the underlying cause and determine whether hydrocephalus is present. If hydrocephalus is present, early delivery and management could be beneficial; however, this protocol depends on maturation of the lungs and stability of the fetus. Postnatal management depends mainly on the cause of the ventriculomegaly. Only patients with obstructive causes and progressive hydrocephalus are candidates for CSF diversion procedures. Conservative measures can be tried in patients whose ventricular diameter is stable and who have no evidence of increased intracranial pressure. The indications for and timing of surgical intervention depend mainly on two factors: (1) the degree and progression of ventriculomegaly, according to findings of clinical examination and neuroimaging follow-up, and (2) the presence or absence of increased intracranial pressure. Surgical treatment of ventriculomegaly in fetuses is similar to that in cases diagnosed after birth and includes different types of CSF reservoir devices, shunts, and endoscopic fenestration [35].

Prognosis

The outcome in affected fetuses depends mainly on the severity of ventriculomegaly and associated anomalies. In isolated ventriculomegaly, the size of the ventricles influences overall outcome, mild isolated ventriculomegaly carries the most favorable prognosis [7,36]. In a study of 176 patients with ventriculomegaly, Gaglioti et al. found that the survival rates were 98%, 80%, and 33% in mild, moderately severe, and severe cases, respectively. The worse outcomes in severe cases are related to underlying structural abnormalities found in 75% of such cases [37]. In a meta-analysis, Pagani et al. found that neurodevelopmental delay was present in only 8% of patients with mild to moderate ventriculomegaly [7]. Conversely, neurodevelopment was normal in only 5%-8% of patients with severe ventriculomegaly [37-39].

## Conclusions

Fetal ventriculomegaly has multiple causes and the developmental outcomes vary. Neuroimaging, including fetal ultrasonography and MRI, help diagnose fetal ventriculomegaly. When it is diagnosed, chromosomal microarray analysis is recommended because it can reveal submicroscopic chromosomal abnormalities that are undetectable in conventional karyotyping. Prenatal management of fetal ventriculomegaly is still limited, and in cases diagnosed both prenatally and after birth, postnatal management includes the use of different types of CSF reservoir devices, shunts, and endoscopic fenestration. The outcome of fetal ventriculomegaly depends mainly on the severity of both ventriculomegaly and any associated anomalies that may be present.
